# Development and validation of a prediction model for tuberculous peritoneal effusion

**DOI:** 10.3389/fmed.2026.1823510

**Published:** 2026-06-19

**Authors:** Libin Liu, Tingting Fang, Qianqian Peng, Hui Wei, Long Cai

**Affiliations:** Centre of Laboratory Medicine, Hangzhou Red Cross Hospital, Hangzhou, Zhejiang, China

**Keywords:** adenosine deaminase, ascites total protein, diagnostic nomogram, prediction model, tuberculous peritoneal effusion

## Abstract

**Purpose:**

The aim of this study was to develop and validate a nomogram for diagnosing tuberculous peritoneal effusion (TPE).

**Patients and methods:**

This was a retrospective, single-center study. We collected data on basic characteristics, laboratory tests, and clinical information from consecutive patients with TPE and non-tuberculous peritoneal effusion (non-TPE) using an electronic medical record database. The data were randomly divided into a training group and a validation group at a 7:3 ratio. Variables were selected using LASSO regression, and a predictive model was established using multivariable stepwise logistic regression to construct the diagnostic nomogram. The diagnostic performance of the nomogram was evaluated using ROC curves, calibration curves, and decision curve analysis (DCA).

**Results:**

A total of 351 patients were included in the study, with 245 in the training group and 106 in the validation group. Age, fever, ascites adenosine deaminase (ADA), ascites carcinoembryonic antigen (CEA), ascites total protein (TP), serum CEA, and serum creatinine (Cr) were identified as independent predictors. Based on these seven variables, we developed a diagnostic nomogram. The area under the curve (AUC) of the nomogram model was 0.974 for the training group and 0.955 for the validation group. The calibration curves showed good agreement, and DCA indicated that the model provides the greatest net benefit within the threshold probability range for both the training and validation cohorts. The analysis of the model’s probability rationality demonstrated that the diagnostic performance of the nomogram was superior to that of any single indicator.

**Conclusion:**

We developed and validated a nomogram model for diagnosing TPE based on seven clinical parameters. This model demonstrates excellent diagnostic performance in distinguishing TPE from non-TPE cases and provides a solid foundation for clinicians to formulate diagnostic strategies.

## Introduction

Tuberculosis remains the leading infectious disease causing the most deaths globally. According to the World Health Organization report, approximately 10.6 million people were infected with tuberculosis in 2022, with 1.3 million deaths resulting from the disease. Extrapulmonary tuberculosis cases have increased and now account for about 15% of all tuberculosis cases (1). TPE is a common clinical manifestation of extrapulmonary tuberculosis. Peritoneal effusion refers to the pathological accumulation of fluid in the abdominal cavity, and tuberculous peritonitis is one of the major causes of TPE, typically occurring as a secondary infection due to hematogenous spread from pulmonary tuberculosis lesions (2, 3). TPE can present in various forms, complicating clinical diagnosis and making it easily confused with other causes of abdominal effusion, such as liver cirrhosis and neoplastic diseases (2, 4). If not diagnosed and treated promptly, it can lead to local complications such as adhesions and subacute bowel obstruction, and may even result in systemic complications and death. Therefore, early differentiation of the type of effusion is crucial for accurate diagnosis and effective treatment of the disease.

Traditionally, the diagnosis of TPE relies on the culture of *mycobacterium tuberculosis* and acid-fast bacilli (AFB) testing. While these methods are highly accurate, they face several challenges, including lower sensitivity, longer processing times, complex procedures, and delayed results (5). Laparoscopy allows for direct peritoneal biopsy for histopathological and microbiological examination. This technique holds significant value in diagnosing TPE (6); however, laparoscopic biopsy is invasive, costly, and requires specialized surgical expertise, which limits its widespread application. Recent advancements in molecular biology and immunology have introduced new diagnostic methods, such as Xpert and interferon-gamma release assays (IGRAs), which show potential advantages in diagnosing TPE. Nevertheless, these methods still face challenges related to insufficient sensitivity or specificity in differentiating TPE from other causes of peritoneal effusion (7–9). Therefore, identifying and validating new biomarkers or diagnostic strategies to enhance the early diagnosis of TPE is crucial for mitigating its impact on both individual health and public health.

Through a retrospective analysis of clinical data from inpatients at our hospital, we constructed a nomogram diagnostic prediction model that significantly improves the ability to distinguish TPE from non-TPE. This model can help clinicians, especially those in primary care settings, make early diagnoses and formulate treatment strategies.

## Materials and methods

We consecutively enrolled hospitalized patients from Hangzhou Red Cross Hospital in Zhejiang Province between November 2013 and April 2024. All patient data were obtained from the hospital’s electronic medical record system. A total of 351 patients were included in the study, comprising 128 cases of TPE and 223 cases of non-TPE. Among the 128 TPE patients in this study, 106 (82.8%) were confirmed cases, while 22 (17.2%) were diagnosed based solely on clinical response. The non-TPE cases mainly include malignant peritoneal effusion (such as abdominal cancer, peritoneal cancer, liver cancer, colon cancer, ovarian cancer, gastric cancer, pancreatic cancer, and other cancers) and benign peritoneal effusion (such as cirrhosis, heart failure, renal failure, bacterial peritonitis, and other benign conditions) (Supplementary Table S1). Patients included in the study met the following criteria: (1) Complete ascites test results; (2) Detailed hospitalization records. Exclusion criteria: (1) Substantial missing laboratory data; (2) Ascites of unknown etiology; (3) Age under 18 years. The main objective of this study is to develop a nomogram-based diagnostic prediction model to accurately differentiate between TPE and non-TPE. This retrospective study was conducted without personal or commercial interests. It was approved by the Ethics Committee of Hangzhou Red Cross Hospital, which granted an exemption from informed consent (Ethical Application Ref: 2024YS085). The research adhered to the Declaration of Helsinki, and the team ensured the confidentiality of all patient data in the electronic medical record system.

### Diagnostic criteria for TPE and non-TPE

The criteria for diagnosing TPE are as follows: (1) Positive acid-fast bacilli staining, culture, or molecular testing in peritoneal tissue or ascitic fluid; (2) Granulomatous inflammation in peritoneal organ biopsy, or positive acid-fast bacilli staining, culture, or molecular testing; (3) Granulomatous inflammation in peritoneal biopsy with an effective response to anti-tuberculosis treatment. Non-TPE includes malignant peritoneal effusion (MPE) and benign peritoneal effusion (BPE): If cancer cells are detected in cytology smears, cell blocks, or peritoneal biopsy, the patient is diagnosed with MPE; If the cause is known and improves or resolves with treatment, the patient is diagnosed with BPE.

### Data collection

This study obtained the following clinical and laboratory data from the clinical electronic record system: age, gender, smoking history, alcohol history, history of tuberculosis, fever symptoms, and routine and biochemical indicators of ascitic fluid (ADA, CEA, alpha-fetoprotein [AFP], carbohydrate antigen 125 [CA125], carbohydrate antigen 15–3 [CA153], carbohydrate antigen 19–9 [CA199], lactate dehydrogenase [LDH], total protein [TP]); and blood routine and biochemical indicators (white blood cells [WBC], neutrophil count [GR], lymphocyte count [LY], hemoglobin [Hb], creatinine [Cr], alanine aminotransferase [ALT], aspartate aminotransferase [AST], C-reactive protein [CRP], ADA, LDH, TP, albumin [Alb], CEA, AFP, CA125, CA153, CA199). Variables included are quantitative data. If the test item has a reference range, the quantitative data are converted to binary data; for quantitative data without a reference range, no conversion is made. Among the 351 patients, 6 (1.7%) lacked test results for blood CEA, AFP, CA125, CA15-3, and CA199; 17 (4.8%) lacked blood ADA results. The missing data accounted for <5%.

### Statistical analysis

First, variables with a missing rate below 5% in the enrolled patient data were imputed using median imputation. The complete dataset was then randomly split into training and validation sets at a 7:3 ratio for model development and performance testing, respectively. For data with skewed distributions, we used the median (M) and quartiles (Q1, Q3) to describe central tendency and dispersion, applying the Mann–Whitney U test or Kruskal-Wallis test for group comparisons. For count data, we reported the number of cases (*n*) and percentages (%), using the Chi-square test or Fisher’s exact test for analysis. LASSO regression was employed for variable selection and data dimensionality reduction, with final model selection done via backward stepwise selection based on the Akaike information criterion. Model performance was assessed with receiver operating characteristic curves, calibration plots, and decision curve analysis (DCA). The compare groups package handled baseline description and difference analysis; the glmnet package was used for LASSO regression; the glm package for multifactor logistic regression; the ggROC package for discrimination analysis; the rms package for calibration and nomograms; the ResourceSelection package for Hosmer–Lemeshow testing; and the rmda package for DCA curves. Decision tree uses the rpart package; random forest uses the randomForest package; XGBoost uses the xgboost package; SVM uses the e1071 package, kNN uses the kknn package; LightGBM uses the lightgbm package. The comparative analysis of the discriminative ability of the nomogram was performed using the pROC package. Statistical analyses were conducted using R software (version 4.2.1) and DCPM (V4.01, Jingding Medical Technology Co., Ltd.).

## Results

**Figure 1:**
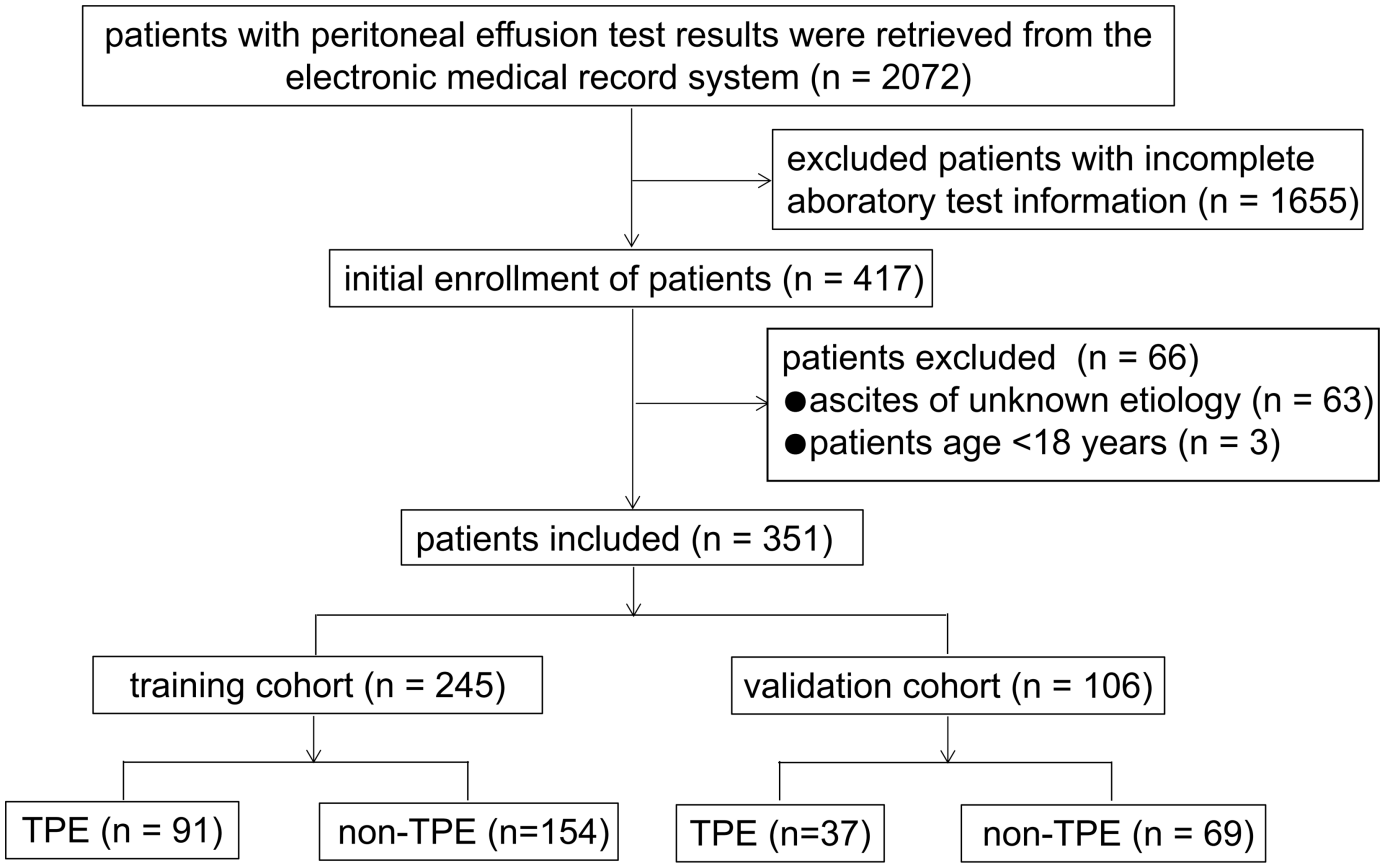
The entry rules of study population. TPE, tuberculous peritoneal effusion; non-TPE, non-tuberculous peritoneal effusion.

From November 2013 to April 2024, our hospital tested 2,072 ascitic fluid samples. First, 1,665 patients with a large amount of missing laboratory data were excluded according to the established criteria. After initial screening, 417 cases were preliminarily included. We then excluded 66 cases: 63 with unclear diagnoses and 3 who were under 18 years old. Ultimately, 351 patients were included in the study. The data were randomly divided into training (*n* = 245) and validation (*n* = 106) groups in a 7:3 ratio. In the training group, there were 91 TPE patients and 154 non-TPE patients, while in the validation group, there were 37 TPE patients and 69 non-TPE patients (Figure 1). The incidence of tuberculous peritoneal effusion was 37.14% (91/245) in the training group and 34.91% (37/106) in the validation group.

### Basic characteristics of the study population

When comparing the general characteristics of the training cohort and the validation cohort, no statistically significant differences were found (*p* = 0.780) (Supplementary Table S2). In the training cohort, comparisons between the TPE group and the non-TPE group revealed significant differences in age, fever symptoms, alcohol history, tuberculosis history, and routine and biochemical ascitic fluid markers (ADA, CEA, AFP, CA19-9, hsCRP, LDH, TP), as well as routine and biochemical blood markers (WBC, LY, Cr, ALT, AST, LDH, TP, CEA, AFP, CA19-9). All these differences were statistically significant (*p* < 0.05) (Table 1).

**Table 1:** Comparison of baseline characteristics between training cohort and validation cohort.

Variables	Training cohort		*p*-value	Validation cohort		*p*-value
	Non-TPE (*N* = 154)	TPE (*N* = 91)		Non-TPE (*N* = 69)	TPE (*N* = 37)	
Age M (Q1, Q3)	72.00 [62.00, 81.00]	43.00 [26.00, 65.00]	<0.001	71.00 [62.00, 83.00]	45.00 [26.00, 68.00]	<0.001
Gender (male), *n* (%)	75 (48.70%)	36 (39.56%)	0.209	52 (75.36%)	23 (62.16%)	0.230
Fever (*n*%)	14 (9.09%)	40 (43.96%)	<0.001	2 (2.90%)	15 (40.54%)	<0.001
Smoking (*n*%)	42 (27.27%)	19 (20.88%)	0.334	21 (30.43%)	8 (21.62%)	0.458
Alcohol (*n*%)	40 (25.97%)	12 (13.19%)	0.028	20 (28.99%)	4 (10.81%)	0.059
History of TB (*n*%)	9 (5.84%)	14 (15.38%)	0.025	1 (1.45%)	2 (5.41%)	0.278
Ascites.ADA (*n*%)			<0.001			<0.001
<24 U/L	146 (94.81%)	25 (27.47%)		65 (94.20%)	11 (29.73%)	
≥24 U/L	8 (5.19%)	66 (72.53%)		4 (5.80%)	26 (70.27%)	
Ascites.CEA (*n*%)			<0.001			0.003
<9.00 ug/L	102 (66.23%)	87 (95.60%)		49 (71.01%)	36 (97.30%)	
≥9.00 ug/L	52 (33.77%)	4 (4.40%)		20 (28.99%)	1 (2.70%)	
Ascites.AFP (*n*%)			0.008			0.295
<20.00 ug/L	143 (92.86%)	91 (100.00%)		65 (94.20%)	37 (100.00%)	
≥20.00 ug/L	11 (7.14%)	0 (0.00%)		4 (5.80%)	0 (0.00%)	
Ascites.CRP (*n*%)			0.011			0.004
<3.30 mg/L	39 (25.32%)	10 (10.99%)		13 (18.84%)	0 (0.00%)	
≥3.30 mg/L	115 (74.68%)	81 (89.01%)		56 (81.16%)	37 (100.00%)	
Ascites.LDH (*n*%)			<0.001			<0.001
<245 U/L	102 (66.23%)	35 (38.46%)		52 (75.36%)	14 (37.84%)	
≥245 U/L	52 (33.77%)	56 (61.54%)		17 (24.64%)	23 (62.16%)	
Ascites.CA125 (*n*%)			–			0.349
<35.00 kU/L	0	0		0 (0.00%)	1 (2.70%)	
≥35.00 kU/L	154 (100.00%)	91 (100.00%)		69 (100.00%)	36 (97.30%)	
Ascites.CA153 (*n*%)			0.224			0.256
<45.00 kU/L	135 (87.66%)	85 (93.41%)		62 (89.86%)	36 (97.30%)	
≥45.00 kU/L	19 (12.34%)	6 (6.59%)		7 (10.14%)	1 (2.70%)	
Ascites.CA199 (*n*%)			<0.001			0.002
<37.00 kU/L	93 (60.39%)	83 (91.21%)		48 (69.57%)	36 (97.30%)	
≥37.00 kU/L	61 (39.61%)	8 (8.79%)		21 (30.43%)	1 (2.70%)	
Ascites.TP M (Q1, Q3)	28.45 [13.83, 41.05]	52.00 [43.65, 58.00]	<0.001	29.60 [17.90, 40.50]	51.00 [46.50, 55.50]	<0.001
Serum.CEA (*n*%)			<0.001			0.004
<5.00 ug/L	106 (68.83%)	89 (97.80%)		53 (76.81%)	37 (100.00%)	
≥5.00 ug/L	48 (31.17%)	2 (2.20%)		16 (23.19%)	0 (0.00%)	
Serum.Alb (*n*%)			0.609			0.72
≥40 g/L	8 (5.19%)	7 (7.69%)		4 (5.80%)	2 (5.41%)	
<40 g/L	146 (94.81%)	84 (92.31%)		65 (94.20%)	35 (94.59%)	
Bood.WBC (*n*%)			0.035			0.014
<9.5 × 10^9/L	128 (83.12%)	85 (93.41%)		53 (76.81%)	36 (97.30%)	
≥9.5 × 10^9/L	26 (16.88%)	6 (6.59%)		16 (23.19%)	1 (2.70%)	
Serum.ALT (*n*%)			0.003			0.333
<40 U/L	113 (73.38%)	82 (90.11%)		55 (79.71%)	33 (89.19%)	
≥40 U/L	41 (26.62%)	9 (9.89%)		14 (20.29%)	4 (10.81%)	
Serum.AST (*n*%)			0.003			0.144
<35 U/L	79 (51.30%)	65 (71.43%)		41 (59.42%)	28 (75.68%)	
≥35 U/L	75 (48.70%)	26 (28.57%)		28 (40.58%)	9 (24.32%)	
Serum.Cr (*n*%)			<0.001			0.004
<111.0 umol/L	115 (74.68%)	89 (97.80%)		50 (72.46%)	36 (97.30%)	
≥111.0 umol/L	39 (25.32%)	2 (2.20%)		19 (27.54%)	1 (2.70%)	
Serum.AFP (*n*%)			0.002			0.089
<20.00 ug/L	141 (91.56%)	91 (100.00%)		63 (91.30%)	37 (100.00%)	
≥20.00 ug/L	13 (8.44%)	0 (0.00%)		6 (8.70%)	0 (0.00%)	
Bood.LY (*n*%)			0.046			0.184
≥1.00 × 10^9/L	50 (32.47%)	18 (19.78%)		25 (36.23%)	8 (21.62%)	
<1.00 × 10^9/L	104 (67.53%)	73 (80.22%)		44 (63.77%)	29 (78.38%)	
Bood.CRP (*n*%)			0.664			0.089
<3.30 mg/L	13 (8.44%)	10 (10.99%)		6 (8.70%)	0 (0.00%)	
≥3.30 mg/L	141 (91.56%)	81 (89.01%)		63 (91.30%)	37 (100.00%)	
Serum.LDH (*n*%)			0.028			0.545
<245 U/L	87 (56.49%)	65 (71.43%)		43 (62.32%)	26 (70.27%)	
≥245 U/L	67 (43.51%)	26 (28.57%)		26 (37.68%)	11 (29.73%)	
Serum.CA125 (*n*%)			0.09			0.667
<35.00 kU/L	13 (8.44%)	2 (2.20%)		11 (15.94%)	4 (10.81%)	
≥35.00 kU/L	141 (91.56%)	89 (97.80%)		58 (84.06%)	33 (89.19%)	
Serum.CA153 (*n*%)			0.175			0.322
<45.00 kU/L	134 (87.01%)	85 (93.41%)		60 (86.96%)	35 (94.59%)	
≥45.00 kU/L	20 (12.99%)	6 (6.59%)		9 (13.04%)	2 (5.41%)	
Serum.CA199 (*n*%)			<0.001			0.003
<37.00 kU/L	90 (58.44%)	81 (89.01%)		49 (71.01%)	36 (97.30%)	
≥37.00 kU/L	64 (41.56%)	10 (10.99%)		20 (28.99%)	1 (2.70%)	
Serum.ADA (*n*%)			0.929			0.128
<24.0 U/L	130 (84.42%)	78 (85.71%)		60 (86.96%)	27 (72.97%)	
≥24.0 U/L	24 (15.58%)	13 (14.29%)		9 (13.04%)	10 (27.03%)	
Bood.Hb (*n*%)			0.062			0.619
Male ≥120 g/L, Female ≥110 g/L	45 (29.22%)	38 (41.76%)		25 (36.23%)	16 (43.24%)	
Male <120 g/L, Female <110 g/L	109 (70.78%)	53 (58.24%)		44 (63.77%)	21 (56.76%)	
Bood.GR (*n*%)			0.969			0.272
≥1.80 × 10^9/L	144 (93.51%)	86 (94.51%)		65 (94.20%)	32 (86.49%)	
<1.80 × 10^9/L	10 (6.49%)	5 (5.49%)		4 (5.80%)	5 (13.51%)	
Serum.TP (*n*%)			0.012			0.237
≥65 g/L	53 (34.42%)	47 (51.65%)		24 (34.78%)	18 (48.65%)	
<65 g/L	101 (65.58%)	44 (48.35%)		45 (65.22%)	19 (51.35%)	

Values are numbers (%) or medians (interquartile ranges).

### Variable screening and model building

**Figure 2:**
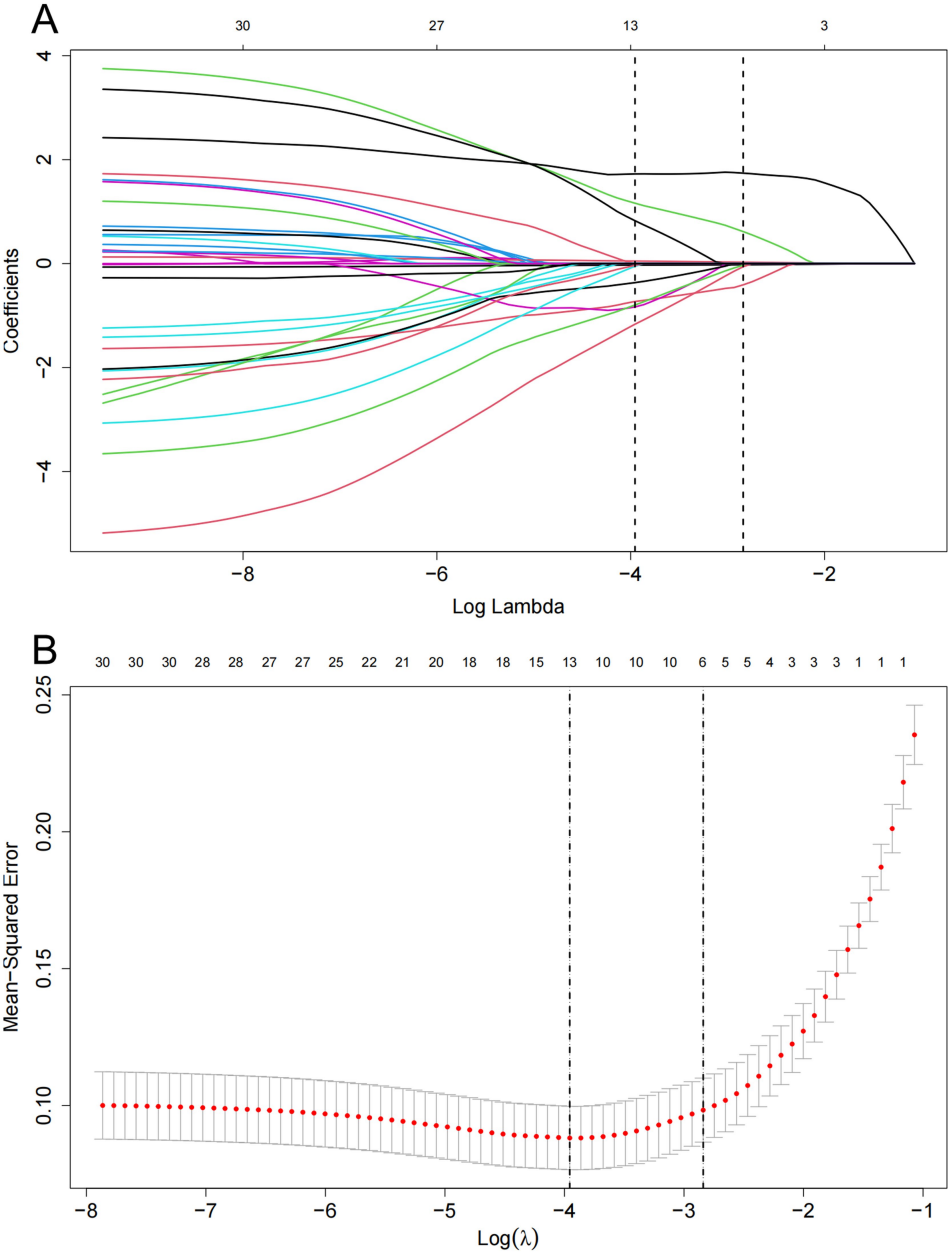
Features selection using the LASSO binary logistic regression model. **(A)** Regression coefficient path diagram; **(B)** cross-validation plot. Ten-fold cross-validation was performed, and seven non-zero coefficient variables were selected based on the Lambda.1se criterion.

**Figure 3:**
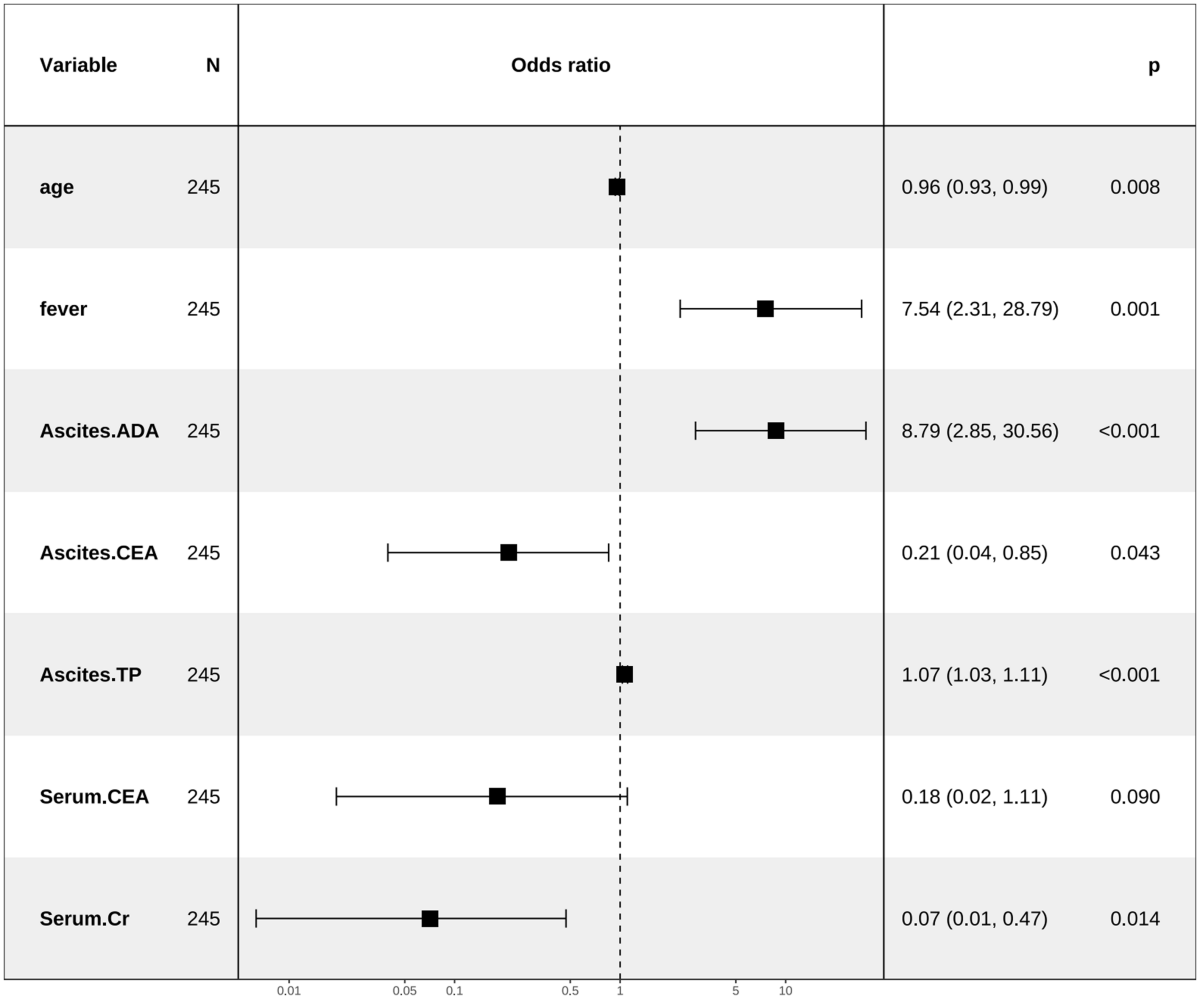
Forest plot of multivariate analysis for predicting tuberculous peritoneal effusion. Variables were selected using backward stepwise regression (AIC criterion) based on the training set.

**Figure 4:**
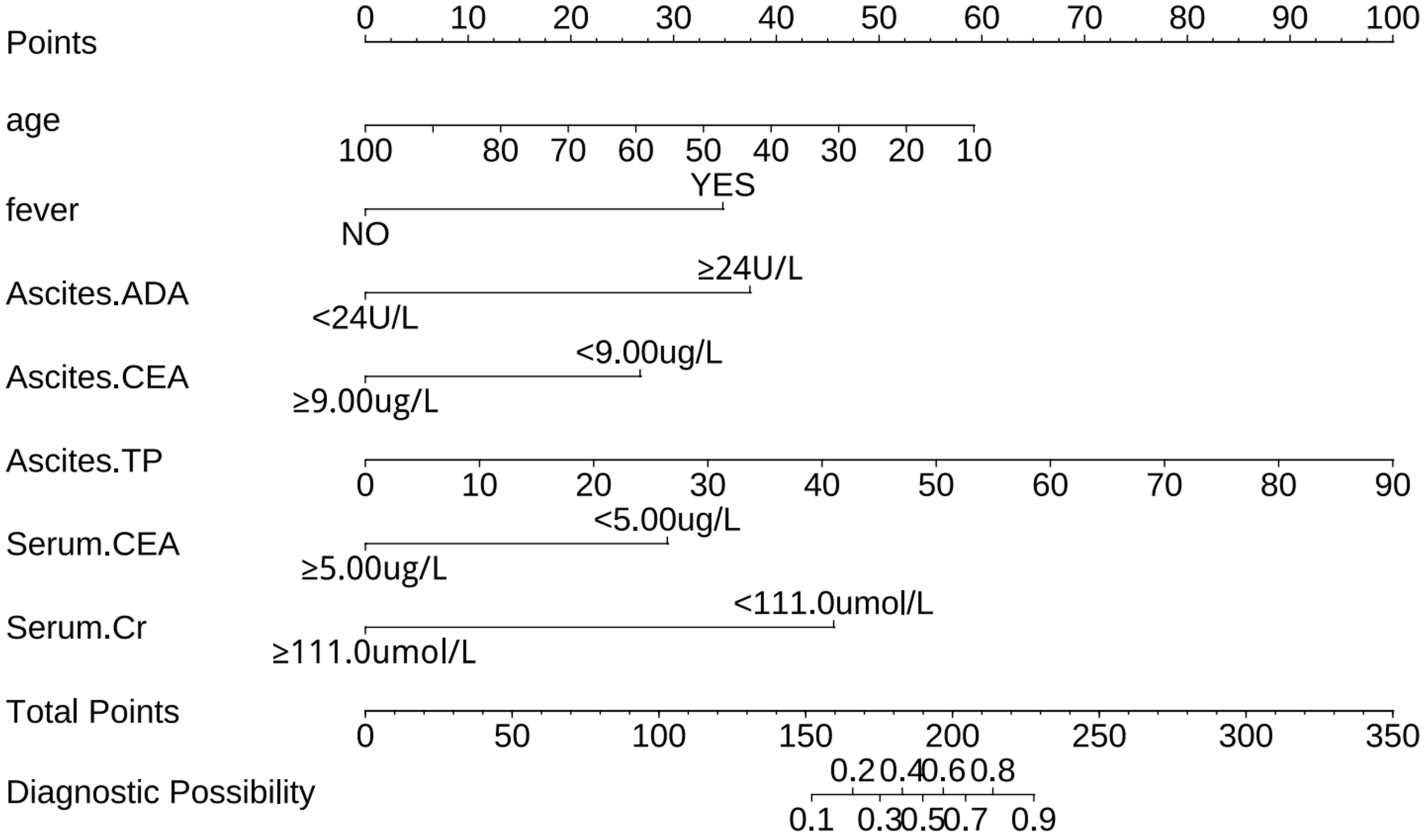
Diagnostic nomogram for predicting tuberculous peritoneal effusion. The score for each variable is linearly transformed from the regression coefficient, and the total score corresponds to the predicted probability. A clinical threshold of 0.48 is recommended.

Using LASSO regression, we selected 7 features from the 32 variables in the training cohort based on the Lambda.Se criterion (age, fever, ascites-ADA, ascites-CEA, ascites-TP, serum-CEA, serum-Cr) (Figures 2A,B). These variables were then included in a multivariate logistic analysis. By applying a backward stepwise selection process with the Akaike Information Criterion, we developed a clinical prediction model incorporating these seven variables and created forest plots and diagnostic nomograms (Figures 3, 4).

### Validation of diagnostic nomogram

**Figure 5:**
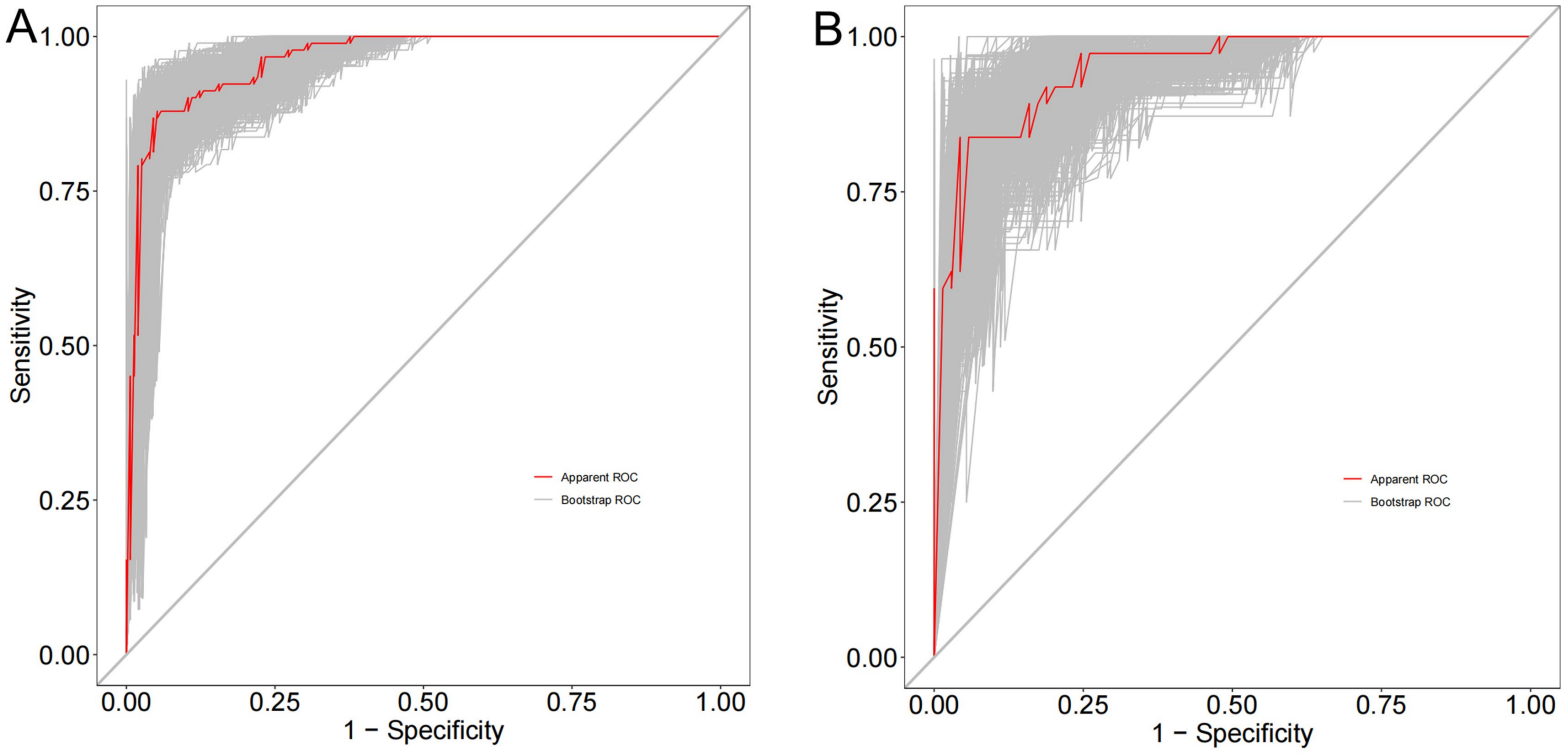
Receiver operating curve for the nomogram was measured by bootstrapping for 500 repetitions. **(A)** training cohort; **(B)** validation cohort. The AUC for the training set is 0.974 (95% CI: 0.958–0.989) and for the validation set is 0.955 (95% CI: 0.920–0.990), both calculated using 500 bootstrap resamples (DeLong method).

**Figure 6:**
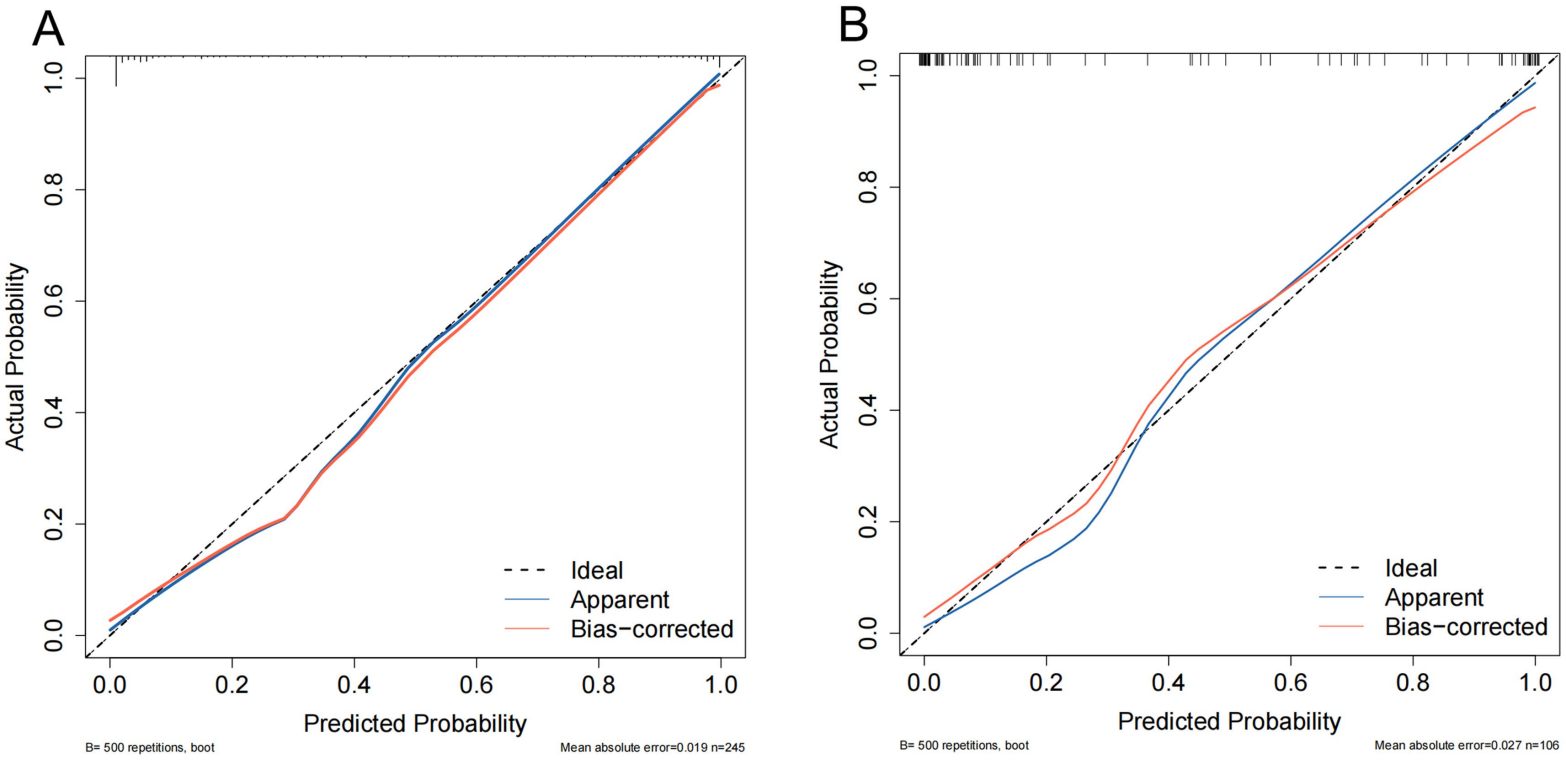
Calibration curve using bootstraps sampling 500 for predicted probability of the nomogram. **(A)** Training cohort; **(B)** validation cohort. Calibration curves were bias-corrected using 500 bootstrap resamples. The Hosmer–Lemeshow test yielded *p* values of 0.578 (training) and 0.311 (validation).

**Figure 7:**
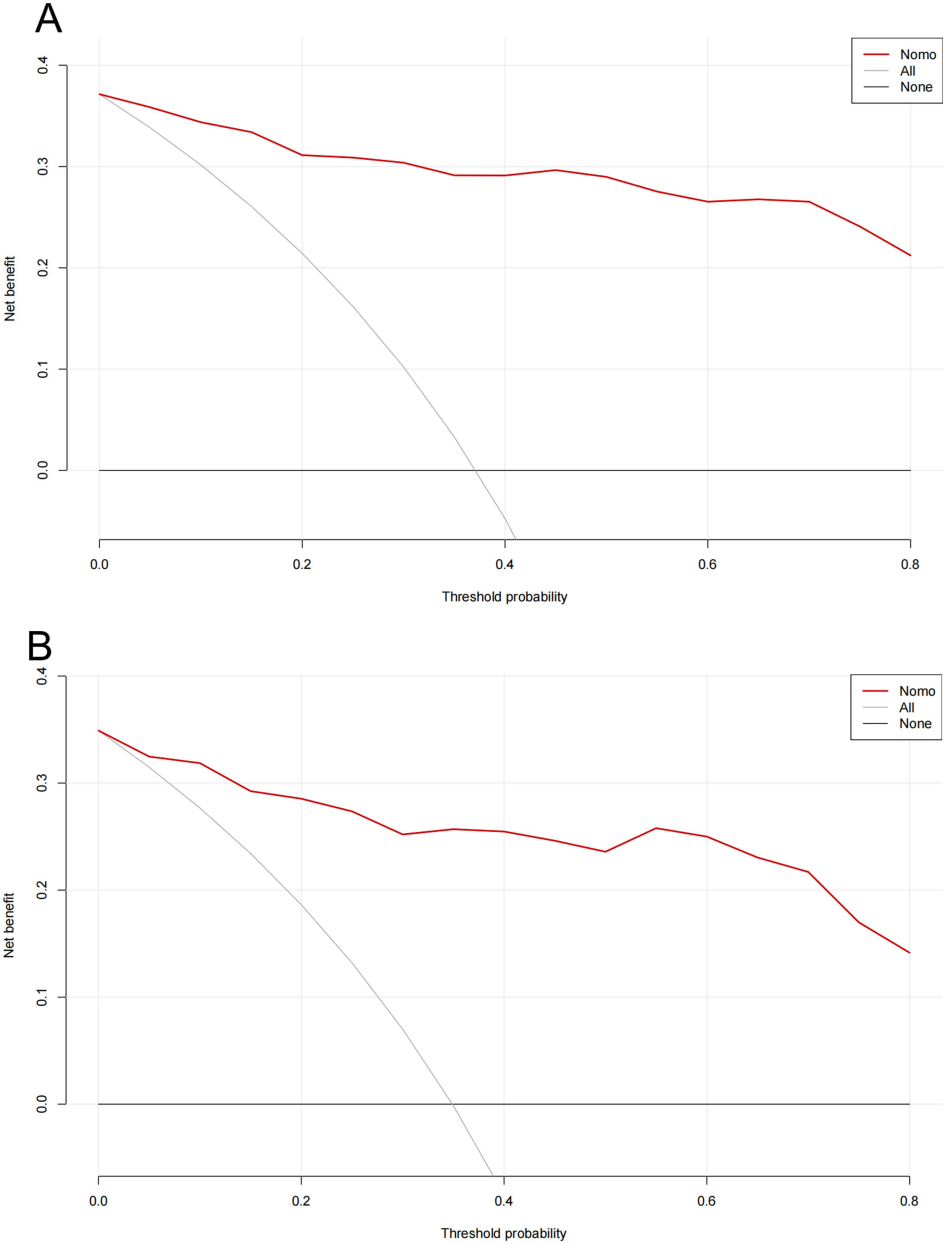
Decision curve analysis using bootstraps sampling 500 for the prediction model. **(A)** training cohort; **(B)** validation cohort. Decision curve analysis was performed using 5-fold cross-validation and 200 bootstrap resamples. The net benefit was greatest at threshold probability ranges of 0.6–100% (training) and 1.3–100% (validation).

Based on these seven variables, we developed a diagnostic model for predicting tuberculous peritoneal effusion. The AUC values for the training cohort and validation cohort were 0.974 (95% CI: 0.958–0.989) and 0.955 (95% CI: 0.920–0.990), respectively. The discrimination analysis showed that the nomogram had a strong ability to identify tuberculous peritoneal effusion (Figures 5A,B). The calibration analysis demonstrated good consistency between predicted and observed values for tuberculous peritoneal effusion (Figures 6A,B). The Hosmer–Lemeshow test indicated that the model’s predicted risks were well aligned with actual risks (training cohort: *χ*^2^ = 6.622, *p* = 0.578; validation cohort: *χ*^2^ = 9.382, *p* = 0.311). Finally, DCA showed that the model achieved the greatest net benefit within the threshold probability range for both the training cohort and the validation cohort (Figures 7A,B). The study suggests that clinical intervention within this probability range is likely to benefit patients.

### Comparison of the diagnostic nomogram with other individual indicators

**Figure 8:**
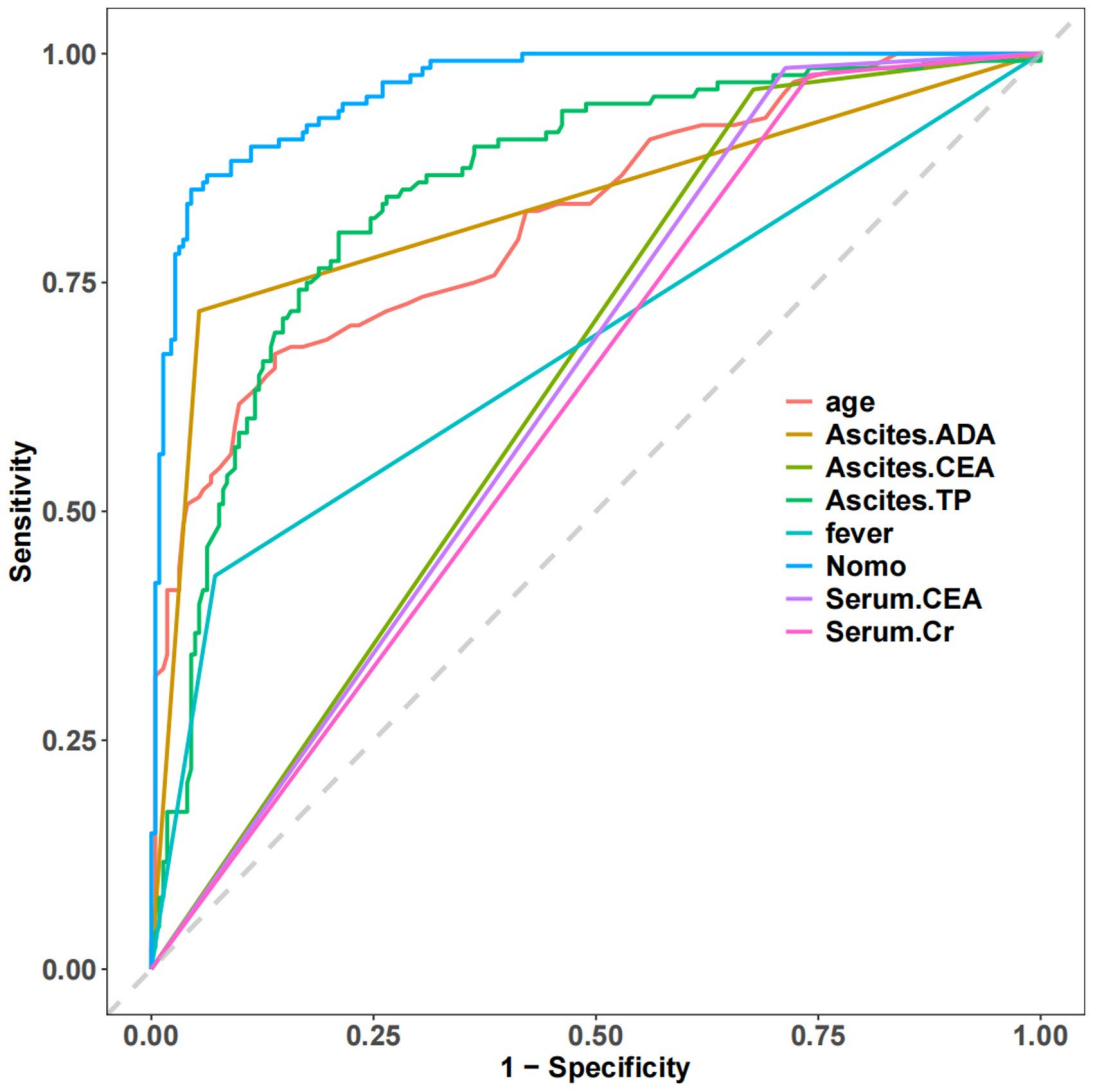
Comparison of diagnostic performance between nomograms and other individual indicators. Pairwise comparisons of AUCs were performed using the DeLong test. The nomogram was significantly superior to all single indicators (*p* < 0.05).

We compared the diagnostic performance of our developed nomogram with that of other individual indicators (age, fever, ascites-ADA, ascites-CEA, ascites-TP, serum-CEA, serum-Cr) in predicting tuberculous peritoneal effusion. ROC analysis showed that the diagnostic efficacy of the nomogram was superior to that of the other single indicators (Figure 8).

### Comparison of diagnostic performance between nomogram and multiple machine learning models in a validation cohort

**Figure 9:**
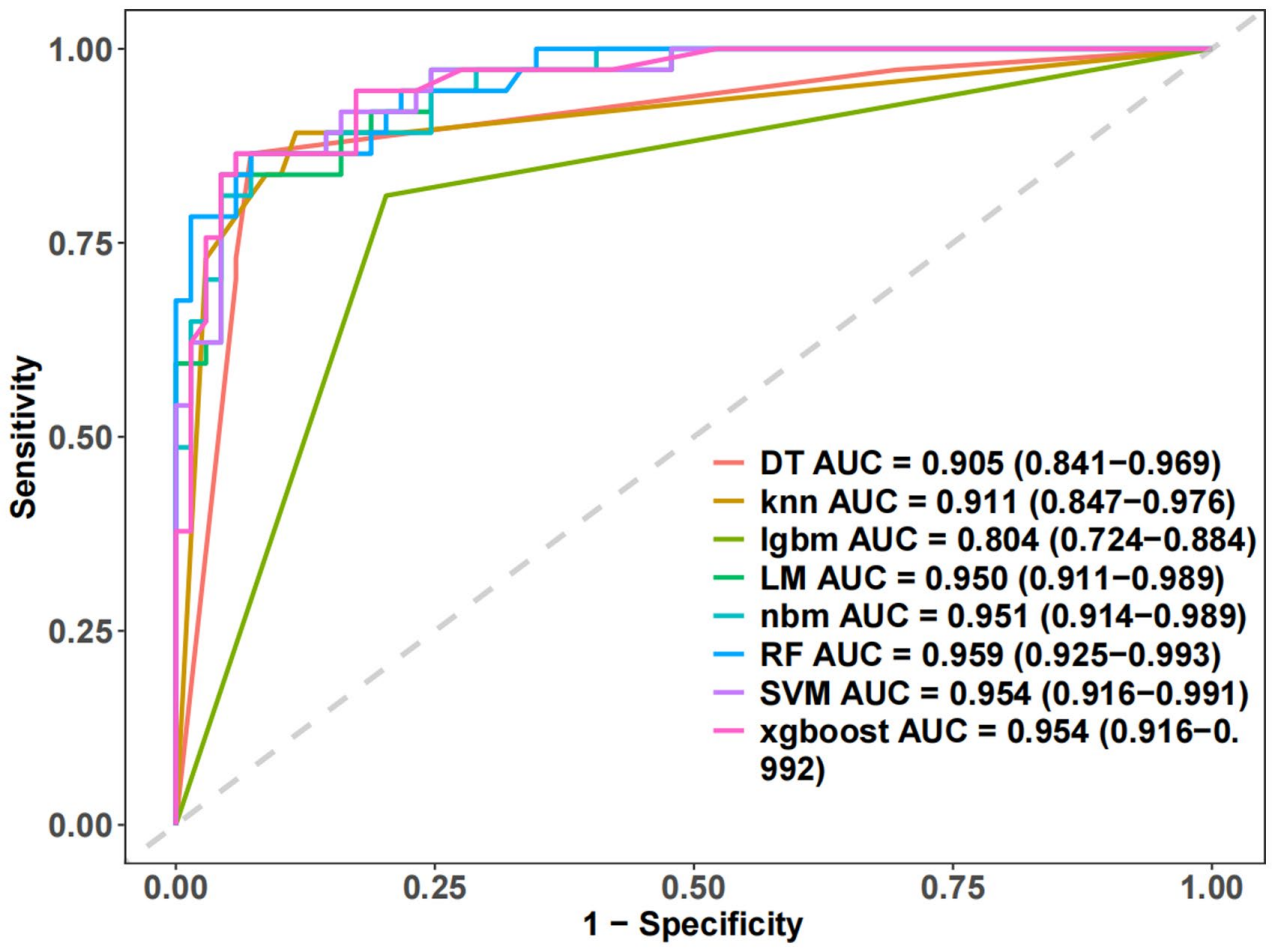
Comparison of diagnostic performance between nomogram and multiple machine learning models. RF, random forest; SVM, support vector machine; nbm, naïve Bayes; Knn, k-nearest neighbors; DT, decision tree; Lgbm, LightGBM; LM, logistic regression model.

To evaluate the differences in diagnostic performance between a nomogram (logistic regression) and mainstream machine learning methods, we compared seven models in the validation cohort: Random Forest (RF), XGBoost, Support Vector Machine (SVM), Naive Bayes (NBM), K-Nearest Neighbors (KNN), Decision Tree (DT), and LightGBM (LGBM). The results showed that RF achieved the highest AUC (0.959), slightly higher than that of the nomogram (0.950). The AUCs of XGBoost, SVM, and NBM were 0.954, 0.954, and 0.951, respectively; those of KNN and DT were 0.911 and 0.905; and LightGBM had the lowest AUC (0.804) (Figure 9). Delong tests indicated that the differences in AUC between RF, XGBoost, SVM and the nomogram were not statistically significant (*p* = 0.738, 0.882, and 0.887, respectively), whereas LightGBM was significantly lower than the nomogram (*p* = 0.002; see Supplementary Table S3). In summary, while maintaining optimal interpretability and ease of use, the nomogram achieved diagnostic performance comparable to that of the machine learning methods.

## Discussion

Early diagnosis and prompt treatment of TPE patients are crucial for preventing severe complications such as intestinal adhesions, bowel obstruction, and the dissemination of infection. Despite the availability of various diagnostic methods, distinguishing between TPE and non-TPE remains challenging in clinical practice. The gold standard for diagnosing TPE is the identification of AFB in ascitic fluid or peritoneal biopsy samples, or the growth of *Mycobacterium tuberculosis*. However, the sensitivity of AFB detection in ascitic fluid is only 3%, and the sensitivity through the culture of *Mycobacterium tuberculosis* improves marginally to 34% (2). Although IGRA shows higher sensitivity, its specificity remains inadequate (7). Many studies have explored the value of ADA in diagnosing TPE, but the diagnostic thresholds for ADA vary across studies, and its diagnostic efficacy can be affected by other bacterial infections or liver cirrhosis (10–13). Thus, seeking new diagnostic strategies is essential for the early diagnosis and timely treatment of TPE patients.

The nomogram, an intuitive clinical prediction model, has gained prominence in the medical field since its initial application by Puppo and Perachino in 1997 for predicting lymph node metastasis in prostate cancer. Over the years of practice and development, nomograms have been increasingly adopted by medical researchers for their unique advantages, providing a scientific basis for clinical decision-making (14–16). In this study, we developed a nomogram-based scoring system to differentiate between TPE and non-TPE. Initially, we included 32 variables covering demographic characteristics, clinical features, and various laboratory test indicators. After applying the LASSO regression to the data, we selected 7 variables (age, fever, ascites-ADA, ascites-CEA, ascites-TP, serum-CEA, serum-Cr) to construct the predictive model. Our nomogram achieved an AUC of 0.955 in the validation set. Although the pooled AUC of ascitic fluid ADA has been reported to reach 0.97, the certainty of this evidence is very low, with substantial heterogeneity and wide variations in ADA cut-off values across studies (21–41 U/L), limiting its clinical stability and generalizability. IGRA has an AUC of approximately 0.91–0.94 but suffers from insufficient specificity. Xpert MTB/RIF shows a sensitivity of only 17.9–25% in ascitic fluid samples, offering limited diagnostic value (7–9, 11). Our model integrates multiple routine indicators, maintaining high diagnostic accuracy while overcoming the limitations of single markers, such as susceptibility to interference, inconsistent cut-off values, and low evidence quality, making it more suitable for primary clinical practice. Clinicians can use this nomogram as follows: Based on the validation set, the optimal probability threshold was determined as 0.48 using the Youden index maximization principle. For each patient, the clinician calculates the total score according to the nomogram and then obtains the corresponding predicted probability: if the probability is ≥0.48, a diagnosis of TPE is favored; if <0.48, TPE is excluded. For example, a 20-year-old female patient presents with fever, ascites ADA ≥ 24 U/L, ascites total protein 63.4 g/L, and all other indicators normal. Her total score is 204 points, which corresponds to a predicted probability of 0.70. Since 0.70 > 0.48, the model predicts TPE, and clinicians may initiate anti-tuberculosis diagnostic treatment or further examinations accordingly (Supplementary Tables S4, S5). In clinical practice, this nomogram can be effectively integrated with existing diagnostic pathways. It can provide immediate risk stratification before the results of microbiological tests (e.g., culture, Xpert) are available, and it can enhance diagnostic confidence when radiological findings are atypical. Such multidimensional integration is particularly applicable in resource-limited or primary healthcare settings.

The determination of ADA levels is a rapid, simple, cost-effective, and efficient testing method. ADA is an enzyme that catalyzes the conversion of adenosine to inosine, with its activity significantly higher in T lymphocytes compared to B lymphocytes and is closely related to the degree of T cell differentiation. When stimulated by mycobacterial antigens, T cell activation leads to elevated ADA levels in tuberculous peritonitis (17, 18). In our study, ADA ≥ 24 U/L was identified as an independent risk factor for TPE (OR = 8.79). Burgess et al. suggested that ADA levels exceeding 30 U/L could be a useful biomarker for differentiating between TPE and non-TPE. Arnoldo Riquelme et al. proposed that the optimal ADA value is 39 U/L. Mayank Mahajan et al. conducted a comparative study of ADA cut-off values and found that the sensitivity and specificity of ADA ≤ 30 U/L and ADA > 30 U/L were similar (9, 10, 19). We chose reference values as the basis for binary classification data conversion; although the predictive ability of a single indicator might not be optimal, this approach facilitates clinical application. Additionally, Donald et al. noted that in the U.S., ascitic ADA is not sensitive for TPE detection, with 59% (10/17) of TPE patients having liver cirrhosis, which introduces bias as ascitic ADA levels in cirrhosis are similar to those in portal hypertensive ascites, thus reducing the diagnostic performance of ascitic ADA (4, 20). Thus, while ADA is valuable for diagnosing TPE, it should be combined with other clinical information for a more accurate diagnosis.

Our study indicates a significant correlation between ascites-TP levels and the occurrence of TPE; higher ascites-TP concentrations are associated with a greater likelihood of TPE. However, it is important to note that Shakil et al. have reported that while ascites-TP levels >25 g/L demonstrate high sensitivity for TPE diagnosis, this level is also present in other types of ascites, including 100% of renal ascites, 100% of cardiac ascites, and up to 95% of peritoneal carcinomatosis ascites (21–23). These findings underscore the limitations of using ascites-TP as a sole diagnostic marker for tuberculosis; while it can provide useful diagnostic clues, it is insufficient on its own for the precise diagnosis of TPE.

In our study, older age was associated with a reduced likelihood of TPE. This finding is consistent with current trends, as TPE can occur at any age but is most prevalent between 35 and 45 years. Research by Dilip K. Das et al. shows that serous malignant effusions are more common in older adults and middle-aged individuals, whereas tuberculous effusions are more frequently observed in younger individuals (24, 25). This observation may be attributed to the increased prevalence of underlying conditions in the elderly, such as tumors, liver cirrhosis, and renal diseases, which are significant contributors to the accumulation of ascitic fluid.

Our study indicates that fever is a crucial indicator for diagnosing TPE. A systematic review of 1,393 patients revealed that approximately 59% of cases presented with fever, reinforcing the close association between fever and TPE (2). It is well established that ascites-CEA, serum-CEA, and serum-Cr levels are significant in assessing the nature of ascites, particularly due to their higher correlation with malignant tumors. Generally, higher levels of carcinoembryonic antigen (CEA) increase the likelihood of malignancy. Serum-Cr levels, closely related to renal function, are important in evaluating kidney disease. Elevated Cr levels may prompt consideration of renal origin for ascites (26, 27). Therefore, when evaluating the cause of ascites, if the CEA or Cr level is low, this may reduce the likelihood of malignancy or nephrogenic disease as the cause of ascites. In this case, other possible causes should be considered, including infectious diseases such as tuberculosis.

In summary, although ADA testing and other single variables show some value in diagnosing TPE, they are also susceptible to interference from other diseases. Therefore, it is crucial to develop a comprehensive predictive model based on multiple indicators for diagnosing TPE. This study is the first to utilize nomograms to differentiate between TPE and non-TPE, with the developed model demonstrating high reliability and accuracy in discriminating between the two. The model incorporates common and diagnostically valuable indicators, offering superior diagnostic performance compared to single variables. These indicators are not only readily available but also cost-effective, and are commonly used as routine tests in most hospitals. Consequently, our model can be conveniently applied in clinical practice across many hospitals, particularly in primary care settings, showing broad applicability.

Notably, compared with various machine learning models, the diagnostic performance of the logistic regression-based nomogram in this study was not inferior (Random Forest, XGBoost, etc., did not show significant superiority). This finding may be related to the limited sample size and the small number of predictors—under conditions of few variables and a moderate sample size, logistic regression generally exhibits good performance and stability. In the future, with the accumulation of multi-center large-sample data, deep learning models could be further explored to improve generalizability. Considering the nomogram’s good interpretability, convenient ease of use, and alignment with guideline recommendations, it demonstrates superior clinical practicality and potential for widespread adoption. Therefore, we chose to retain the original nomogram as the primary prediction model.

This study has the following limitations: First, it is a retrospective, single-center design with a limited sample size, lacks external validation, and precludes more detailed subgroup analyses. Second, only routine serum and ascitic fluid indicators were included; potential biomarkers such as IGRA and IL-2 were not incorporated, and CT/ultrasound imaging data were lacking. Moreover, key indicators used conventional reference ranges rather than optimal thresholds, which, although facilitating generalizability, may reduce diagnostic efficacy. Third, some patients were excluded because they only underwent routine ascitic fluid examination without core indicators such as ADA, resulting in a final cohort that accounted for only a small fraction of the original population, leading to potential systematic differences between included and excluded patients and thus disease spectrum enrichment. Meanwhile, a small number of TPE cases were diagnosed solely based on clinical response to anti-tuberculosis treatment, which, although reflecting real-world practice, introduces diagnostic uncertainty. The above selection bias and diagnostic uncertainty, together with the limited sample size and single-center same-source validation, collectively lead to an overestimation of the model’s AUC (an AUC > 0.95 is unusually high for a clinical prediction model, indicating optimistic bias), and the calibration may also be slightly biased. Therefore, these performance estimates should be interpreted with caution, and independent external validation is urgently needed. Future efforts should focus on prospective, multi-center studies with standardized data collection, larger sample sizes, inclusion of more indicators, and optimization of thresholds to comprehensively assess and correct for biases, thereby improving the model’s generalizability and practical value.

## Conclusion

In this study, we developed a risk prediction nomogram comprising seven variables based on demographic characteristics, clinical presentations, and laboratory tests. This nomogram effectively distinguishes between TPE and non-TPE, providing valuable references for disease management and treatment planning for clinicians. It is especially beneficial in areas where tuberculosis drug susceptibility testing has not yet been implemented, thereby playing a significant role in these regions.

## Data Availability

The original contributions presented in the study are included in the article/Supplementary material, further inquiries can be directed to the corresponding author.
