# Supplementary material for: Development and validation of a prediction model for tuberculous peritoneal effusion
Source: Front Med (Lausanne). 2026 Jun 19;13:1823510. doi: 10.3389/fmed.2026.1823510 (PMC13327908; doi:10.3389/fmed.2026.1823510)
Supplement: Supplementary file 1 [file Table_1.DOCX]

Supplementary table 1: Baseline characteristics of the training group and validation group

| characteristics | total(N=351) | Training group (N=245) | validation group (N=106) |
| --- | --- | --- | --- |
| Age, median M (Q1, Q3) | 65.00 [50.00;78.00] | 65.00 [49.00;77.00] | 67.00 [52.25;79.75] |
| Sex (male, n%) | 186 (52.99) | 111 (45.31) | 75 (70.75) |
| TPE |  |  |  |
| Tuberculous peritonitis (n%) | 128(36.47) | 91(37.14) | 37(34.91) |
| MPE |  |  |  |
| Abdominal cancer (n%) | 14(3.99) | 11(4.49) | 3(2.83) |
| Peritoneal cancer (n%) | 10(2.85) | 9(3.67) | 1(0.94) |
| Liver cancer (n%) | 31(8.83) | 22(8.98) | 9(8.49) |
| Colon cancer (n%) | 9(2.56) | 7(2.86) | 2(1.89) |
| Ovarian cancer (n%) | 10(2.85) | 6(2.45) | 4(3.77) |
| Gastric cancer (n%) | 20(5.7) | 13(5.31) | 7(6.6) |
| Pancreatic cancer (n%) | 13(3.7) | 7(2.86) | 6(5.66) |
| Other cancers (n%) | 28(7.98) | 16(6.53) | 12(11.32) |
| BPE |  |  |  |
| Cirrhosis (n%) | 48(13.68) | 33(13.47) | 15(14.15) |
| Heart failure (n%) | 14(3.99) | 9(3.67) | 5(4.72) |
| Renal failure (n%) | 10(2.85) | 8(3.27) | 2(1.89) |
| Bacterial peritonitis (n%) | 6(1.71) | 5(2.04) | 1(0.94) |
| Other benign diseases (n%) | 10(2.85) | 8(3.27) | 2(1.89) |

Note: other cancers, Rectal tumor, bladder tumor, kidney tumor, gallbladder tumor, lymphoma, mediastinal tumor, duodenal tumor, prostate tumor, ampullary tumor, cervical tumor, lung tumor, tumor of unknown origin; Other benign diseases, intestinal obstruction, Lymphatic drainage disorder, Liver abscess, Gastrointestinal bleeding disorders, Chronic pancreatitis, Chronic cholecystitis, Ovarian cysts, Eosinophilic peritonitis, Hyperthyroidism liver damage. Abbreviations: TPE, tuberculous peritoneal effusion; MPE, malignant peritoneal effusion; BPE, benign peritoneal effusion.
