# Supplementary material for: Development and validation of a prediction model for tuberculous peritoneal effusion
Source: Front Med (Lausanne). 2026 Jun 19;13:1823510. doi: 10.3389/fmed.2026.1823510 (PMC13327908; doi:10.3389/fmed.2026.1823510)
Supplement: Supplementary file 2 [file Table_2.DOCX]

Supplementary table 2 Comparison of general data between the training group and the validation group

| characteristics | ALL (N=351) | Training group (N=245) | Validation group (N=106) | *P*-value |
| --- | --- | --- | --- | --- |
| Grouped (n%) |  |  |  | 0.780 |
| 0 | 223 (63.53%) | 154 (62.86%) | 69 (65.09%) |  |
| 1 | 128 (36.47%) | 91 (37.14%) | 37 (34.91%) |  |
| Age, M (Q1, Q3) | 65.00 [50.00;78.00] | 65.00 [49.00;77.00] | 67.00 [52.25;79.75] | 0.400 |
| Sex (n%) |  |  |  | <0.001 |
| Female | 165 (47.01%) | 134 (54.69%) | 31 (29.25%) |  |
| Male | 186 (52.99%) | 111 (45.31%) | 75 (70.75%) |  |
| Fever (n%) |  |  |  | 0.254 |
| No | 280 (79.77%) | 191 (77.96%) | 89 (83.96%) |  |
| Yes | 71 (20.23%) | 54 (22.04%) | 17 (16.04%) |  |
| Smoking (n%) |  |  |  | 0.725 |
| No | 261 (74.36%) | 184 (75.10%) | 77 (72.64%) |  |
| Yes | 90 (25.64%) | 61 (24.90%) | 29 (27.36%) |  |
| Alcohol (n%) |  |  |  | 0.877 |
| No | 275 (78.35%) | 193 (78.78%) | 82 (77.36%) |  |
| Yes | 76 (21.65%) | 52 (21.22%) | 24 (22.64%) |  |
| History of TB (n%) |  |  |  | 0.053 |
| No | 325 (92.59%) | 222 (90.61%) | 103 (97.17%) |  |
| Yes | 26 (7.41%) | 23 (9.39%) | 3 (2.83%) |  |
| Ascites.ADA (n%) |  |  |  | 0.817 |
| <24U/L | 247 (70.37%) | 171 (69.80%) | 76 (71.70%) |  |
| ≥24U/L | 104 (29.63%) | 74 (30.20%) | 30 (28.30%) |  |
| Ascites.CEA (n%) |  |  |  | 0.622 |
| <9.00ug/L | 274 (78.06%) | 189 (77.14%) | 85 (80.19%) |  |
| ≥9.00ug/L | 77 (21.94%) | 56 (22.86%) | 21 (19.81%) |  |
| Ascites.AFP (n%) |  |  |  | 0.986 |
| <20.00ug/L | 336 (95.73%) | 234 (95.51%) | 102 (96.23%) |  |
| ≥20.00ug/L | 15 (4.27%) | 11 (4.49%) | 4 (3.77%) |  |
| Ascites.CRP (n%) |  |  |  | 0.111 |
| <3.30mg/L | 62 (17.66%) | 49 (20.00%) | 13 (12.26%) |  |
| ≥3.30mg/L | 289 (82.34%) | 196 (80.00%) | 93 (87.74%) |  |
| Ascites.LDH (n%) |  |  |  | 0.323 |
| <245U/L | 203 (57.83%) | 137 (55.92%) | 66 (62.26%) |  |
| ≥245U/L | 148 (42.17%) | 108 (44.08%) | 40 (37.74%) |  |
| Ascites.CA125 (n%) |  |  |  | 0.302 |
| <35.00kU/L | 1 (0.28%) | 0 (0.00%) | 1 (0.94%) |  |
| ≥35.00kU/L | 350 (99.72%) | 245 (100.00%) | 105 (99.06%) |  |
| Ascites.CA153 (n%) |  |  |  | 0.559 |
| <45.00kU/L | 318 (90.60%) | 220 (89.80%) | 98 (92.45%) |  |
| ≥45.00kU/L | 33 (9.40%) | 25 (10.20%) | 8 (7.55%) |  |
| Ascites.CA199 (n%) |  |  |  | 0.186 |
| <37.00kU/L | 260 (74.07%) | 176 (71.84%) | 84 (79.25%) |  |
| ≥37.00kU/L | 91 (25.93%) | 69 (28.16%) | 22 (20.75%) |  |
| Ascites.TP M(Q1, Q3) | 38.80 [20.60;50.95] | 39.20 [19.80;51.30] | 37.80 [22.58;50.80] | 0.985 |
| Serum.CEA (n%) |  |  |  | 0.307 |
| <5.00ug/L | 285 (81.20%) | 195 (79.59%) | 90 (84.91%) |  |
| ≥5.00ug/L | 66 (18.80%) | 50 (20.41%) | 16 (15.09%) |  |
| Serum.Alb (n%) |  |  |  | 0.938 |
| ≥40g/L | 21 (5.98%) | 15 (6.12%) | 6 (5.66%) |  |
| <40g/L | 330 (94.02%) | 230 (93.88%) | 100 (94.34%) |  |
| Bood.WBC (n%) |  |  |  | 0.568 |
| <9.5×10^9/L | 302 (86.04%) | 213 (86.94%) | 89 (83.96%) |  |
| ≥9.5×10^9/L | 49 (13.96%) | 32 (13.06%) | 17 (16.04%) |  |
| Serum.ALT (n%) |  |  |  | 0.549 |
| <40U/L | 283 (80.63%) | 195 (79.59%) | 88 (83.02%) |  |
| ≥40U/L | 68 (19.37%) | 50 (20.41%) | 18 (16.98%) |  |
| Serum.AST (n%) |  |  |  | 0.320 |
| <35U/L | 213 (60.68%) | 144 (58.78%) | 69 (65.09%) |  |
| ≥35U/L | 138 (39.32%) | 101 (41.22%) | 37 (34.91%) |  |
| Serum.Cr (n%) |  |  |  | 0.741 |
| <111.0umol/L | 290 (82.62%) | 204 (83.27%) | 86 (81.13%) |  |
| ≥111.0umol/L | 61 (17.38%) | 41 (16.73%) | 20 (18.87%) |  |
| Serum.AFP (n%) |  |  |  | 0.903 |
| <20.00ug/L | 332 (94.59%) | 232 (94.69%) | 100 (94.34%) |  |
| ≥20.00ug/L | 19 (5.41%) | 13 (5.31%) | 6 (5.66%) |  |
| Bood.LY (n%) |  |  |  | 0.608 |
| ≥1.00×10^9/L | 101 (28.77%) | 68 (27.76%) | 33 (31.13%) |  |
| <1.00×10^9/L | 250 (71.23%) | 177 (72.24%) | 73 (68.87%) |  |
| Bood.CRP (n%) |  |  |  | 0.340 |
| <3.30mg/L | 29 (8.26%) | 23 (9.39%) | 6 (5.66%) |  |
| ≥3.30mg/L | 322 (91.74%) | 222 (90.61%) | 100 (94.34%) |  |
| Serum.LDH (n%) |  |  |  | 0.672 |
| <245U/L | 221 (62.96%) | 152 (62.04%) | 69 (65.09%) |  |
| ≥245U/L | 130 (37.04%) | 93 (37.96%) | 37 (34.91%) |  |
| Serum.CA125 (n%) |  |  |  | 0.024 |
| <35.00kU/L | 30 (8.55%) | 15 (6.12%) | 15 (14.15%) |  |
| ≥35.00kU/L | 321 (91.45%) | 230 (93.88%) | 91 (85.85%) |  |
| Serum.CA153 (n%) |  |  |  | 0.902 |
| <45.00kU/L | 314 (89.46%) | 219 (89.39%) | 95 (89.62%) |  |
| ≥45.00kU/L | 37 (10.54%) | 26 (10.61%) | 11 (10.38%) |  |
| Serum.CA199 (n%) |  |  |  | 0.060 |
| <37.00kU/L | 256 (72.93%) | 171 (69.80%) | 85 (80.19%) |  |
| ≥37.00kU/L | 95 (27.07%) | 74 (30.20%) | 21 (19.81%) |  |
| Serum.ADA (n%) |  |  |  | 0.614 |
| <24.0U/L | 295 (84.05%) | 208 (84.90%) | 87 (82.08%) |  |
| ≥24.0U/L | 56 (15.95%) | 37 (15.10%) | 19 (17.92%) |  |
| Bood.Hb (n%) |  |  |  | 0.458 |
| Male ≥ 120g/L, Female ≥ 110g/L | 124 (35.33%) | 83 (33.88%) | 41 (38.68%) |  |
| Male < 120g/L, Female < 110g/L | 227 (64.67%) | 162 (66.12%) | 65 (61.32%) |  |
| Bood.GR (n%) |  |  |  | 0.564 |
| ≥1.80×10^9/L | 327 (93.16%) | 230 (93.88%) | 97 (91.51%) |  |
| <1.80×10^9/L | 24 (6.84%) | 15 (6.12%) | 9 (8.49%) |  |
| Serum.TP (n%) |  |  |  | 0.928 |
| ≥65g/L | 142 (40.46%) | 100 (40.82%) | 42 (39.62%) |  |
| <65g/L | 209 (59.54%) | 145 (59.18%) | 64 (60.38%) |  |
