# Supplementary material for: Development and validation of a prediction model for tuberculous peritoneal effusion
Source: Front Med (Lausanne). 2026 Jun 19;13:1823510. doi: 10.3389/fmed.2026.1823510 (PMC13327908; doi:10.3389/fmed.2026.1823510)
Supplement: Supplementary file 3 [file Table_3.DOCX]

Supplementary Table 3. Matrix of P-values for pairwise comparisons of AUCs between different models using the DeLong test.

|  | DT | LM | RF | XGBOOST | svm | KNN | lgbm |
| --- | --- | --- | --- | --- | --- | --- | --- |
| DT | 1 | 0.23885023 | 0.011143856 | 0.043198769 | 0.071184378 | 0.823992627 | 0.054572523 |
| LM | 0.23885023 | 1 | 0.737566209 | 0.881615624 | 0.88678322 | 0.319252407 | 0.001559732 |
| RF | 0.011143856 | 0.737566209 | 1 | 0.699035377 | 0.695586897 | 0.037767443 | 0.000630702 |
| XGBOOST | 0.043198769 | 0.881615624 | 0.699035377 | 1 | 0.986056128 | 0.148200154 | 0.001093196 |
| svm | 0.071184378 | 0.88678322 | 0.695586897 | 0.986056128 | 1 | 0.080083939 | 0.001092649 |
| KNN | 0.823992627 | 0.319252407 | 0.037767443 | 0.148200154 | 0.080083939 | 1 | 0.041455305 |
| lgbm | 0.054572523 | 0.001559732 | 0.000630702 | 0.001093196 | 0.001092649 | 0.041455305 | 1 |
