# Supplementary material for: Development and validation of a prediction model for tuberculous peritoneal effusion
Source: Front Med (Lausanne). 2026 Jun 19;13:1823510. doi: 10.3389/fmed.2026.1823510 (PMC13327908; doi:10.3389/fmed.2026.1823510)
Supplement: Supplementary file 4 [file Table_4.DOCX]

| **age** | | **fever** | | **Ascites.ADA** | | **Ascites.CEA** | | **Ascites.CEA** |  | **Ascites.TP** | | **Serum.CEA** | | **Serum.Cr** | | **Total Points Diagnostic Possibility** | |
| --- | --- | --- | --- | --- | --- | --- | --- | --- | --- | --- | --- | --- | --- | --- | --- | --- | --- |
| **result** | **Score** | **result** | **Score** | **result** | **Score** | **result** | **Score** | **result** | **Score** | **result** | **Score** | **result** | **Score** | **result** | **Score** | **Total Score** | **predict value** |
| 10 | 59 | 0 | 0 | 0 | 0 | 0 | 0 | 0 | 0 | 0 | 0 | 0 | 0 | 0 | 0 | 152 | 0.1 |
| 20 | 53 | 1 | 35 | 1 | 37 | 1 | 27 | 1 | 27 | 10 | 11 | 1 | 29 | 1 | 46 | 166 | 0.2 |
| 30 | 46 |  |  |  |  |  |  |  |  | 20 | 22 |  |  |  |  | 175 | 0.3 |
| 40 | 39 |  |  |  |  |  |  |  |  | 30 | 33 |  |  |  |  | 183 | 0.4 |
| 50 | 33 |  |  |  |  |  |  |  |  | 40 | 44 |  |  |  |  | 190 | 0.5 |
| 60 | 26 |  |  |  |  |  |  |  |  | 50 | 56 |  |  |  |  | 197 | 0.6 |
| 70 | 20 |  |  |  |  |  |  |  |  | 60 | 67 |  |  |  |  | 204 | 0.7 |
| 80 | 13 |  |  |  |  |  |  |  |  | 70 | 78 |  |  |  |  | 214 | 0.8 |
| 90 | 7 |  |  |  |  |  |  |  |  | 80 | 89 |  |  |  |  | 228 | 0.9 |
| 100 | 0 |  |  |  |  |  |  |  |  | 90 | 100 |  |  |  |  |  |  |

Supplementary Material 4. Nomogram Score–Probability Reference Table.
