# Supplementary material for: Development and validation of a prediction model for tuberculous peritoneal effusion
Source: Front Med (Lausanne). 2026 Jun 19;13:1823510. doi: 10.3389/fmed.2026.1823510 (PMC13327908; doi:10.3389/fmed.2026.1823510)
Supplement: Supplementary file 5 [file Table_5.DOCX]

**Clinical Application Example of the Nomogram**

**Patient information:**

Age: 20 years

Sex: Female

Fever: Yes

Ascites ADA: ≥24 U/L (abnormal, coded as 1)

Ascites CEA: <9 μg/L (normal)

Ascites total protein: 63.4 g/L (abnormal, reference <30 g/L)

Serum CEA: <5 μg/L (normal)

Serum Cr: <111 μmol/L (normal)

**Using the nomogram (refer to Nomogram Score.xlsx and Figure 4):**

1. Assign scores to each variable:

Age 20 → 53 points

Fever (yes) → 35 points

Ascites ADA abnormal → 37 points

Ascites CEA normal → 0 points

Ascites TP 63.4 g/L → ~79 points (between 60 g/L → 67 points and 70 g/L → 78 points)

Serum CEA normal → 0 points

Serum Cr normal → 0 points

1. **Calculate total points: 53 + 35 + 37 + 0 + 79 + 0 + 0 = 204 points**
2. **Obtain predicted probability:**

From the Probability in the appendix, the actual predicted probability is 0.70.

1. **Compare with threshold:**

The recommended threshold is 0.48. The patient’s probability (0.70) > 0.48, so the model predicts tuberculous peritoneal effusion (TPE).

1. **Clinical decision:**

Anti-tuberculosis diagnostic treatment or further laparoscopy/biopsy may be initiated for confirmation.
